# A Newly Identified Monoterpenoid-Based Small Molecule Able to Support the Survival of Primary Cultured Dopamine Neurons and Alleviate MPTP-Induced Toxicity *In Vivo*

**DOI:** 10.3390/molecules27238286

**Published:** 2022-11-28

**Authors:** Anastasiia Kotliarova, Alexandra V. Podturkina, Alla V. Pavlova, Daria S. Gorina, Anastasiya V. Lastovka, Oleg V. Ardashov, Artem D. Rogachev, Arseniy E. Izyurov, Alla B. Arefieva, Alexander V. Kulikov, Tatyana G. Tolstikova, Konstantin P. Volcho, Nariman F. Salakhutdinov, Yulia Sidorova

**Affiliations:** 1 Department of Medicinal Chemistry, N. N. Vorozhtsov Novosibirsk Institute of Organic Chemistry, Siberian Branch, Russian Academy of Sciences, Lavrentiev Ave., 9, 630090 Novosibirsk, Russia; 2 Laboratory of Molecular Neuroscience, Institute of Biotechnology, HiLIFe, Viikinkaari 5D, University of Helsinki, 00014 Helsinki, Finland; 3 Faculty of Natural Sciences, Novosibirsk State University, Pirogova, 2, 630090 Novosibirsk, Russia; 4 V. Zelman Institute for Medicine and Psychology, Novosibirsk State University, Pirogova, 2, 630090 Novosibirsk, Russia; 5 Department of Genetic Collections of Neural Disorders, Federal Research Center Institute of Cytology and Genetic, Siberian Branch of Russian Academy of Sciences, 630090 Novosibirsk, Russia

**Keywords:** Parkinson’s disease, MPTP, small molecules, dopamine neurons, tyrosine hydroxylase, Prottremin, epoxydiol, drug development, medicinal chemistry, neurodegeneration

## Abstract

Parkinson’s disease (PD) is the most common age-related movement disorder characterized by the progressive loss of nigrostriatal dopaminergic neurons. To date, PD treatment strategies are mostly based on dopamine replacement medicines, which can alleviate motor symptoms but do not slow down the progression of neurodegeneration. Thus, there is a need for disease-modifying PD therapies. The aim of this work was to evaluate the neuroprotective effects of the novel compound PA96 on dopamine neurons in vivo and in vitro, assess its ability to alleviate motor deficits in MPTP- and haloperidol-based PD models, as well as PK profile and BBB penetration. PA96 was synthesized from (1*R*,2*R*,6*S*)-3-methyl-6-(prop-1-en-2-yl) cyclohex-3-ene-1,2-diol (Prottremin) using the original three-step stereoselective procedure. We found that PA96: (1) supported the survival of cultured näive dopamine neurons; (2) supported the survival of MPP^+^-challenged dopamine neurons in vitro and in vivo; (3) had chemically appropriate properties (synthesis, solubility, etc.); (4) alleviated motor deficits in MPTP- and haloperidol-based models of PD; (5) penetrated the blood–brain barrier in vivo; and (6) was eliminated from the bloodstream relative rapidly. In conclusion, the present article demonstrates the identification of PA96 as a lead compound for the future development of this compound into a clinically used drug.

## 1. Introduction

Parkinson’s disease (PD) is the most common age-related progressive neurodegenerative disorder associated with a significant economic burden on the healthcare system. Indeed, the estimated annual European cost of PD management is EUR 13.9 billion [1], which will increase as the number of people with PD in Europe continues to grow. PD is characterized by the classical motor features, including resting tremor, bradykinesia, rigidity, and postural instability, which are caused by the loss of nigrostriatal dopaminergic (DA) neurons [2]. Despite a long history of PD research, there is still no cure. Symptomatic treatment for PD has been available since the 1960s, but to this day, all the FDA-approved drugs cannot reverse or at least stop the process of DA neuronal death [2,3]. Thus, there is an urgent need for disease-modifying PD therapies.

The potential disease-modifying treatments can be developed based on small molecules that can function as the survival and maintenance agents for both developing and mature neurons [4]. Since pharmacological targets for CNS disorders are located mainly beyond the blood–brain barrier (BBB), these targets can be inaccessible to larger, polar molecules. Particularly, the structure of the BBB prevents the passage of molecules with masses >600 Da, a requirement that ultimately restricts up to 98% of small molecules and almost all biologics [5,6]. Indeed, the ongoing clinical trial data have shown that the majority of the trials were small molecular therapeutic strategies (29 trials, 61.7%) [7].

Previously we identified BBB-penetrating monoterpenoid (1*R*,2*R*,6*S*)-3-methyl-6-(prop-1-en-2-yl)cyclohex-3-ene-1,2-diol (Prottremin), demonstrating anti-PD activity in animal MPTP (1-methyl-4-phenyl-1,2,3,6-tetrahydropyridine), rotenone, 6-hydroxydopamine (6-OHDA), and haloperidol models of PD [8,9,10,11].

The lack of information about the molecular target of Prottremin makes it challenging to search for more active analogs of this compound, forcing the ”trial and error“ method to work. Therefore, all eight stereoisomers of Prottremin were synthesized, and the most active isomer was chosen for its ability to reduce the symptoms of hypokinesia in the MPTP-based mouse model of acute PD [9]. It was demonstrated that removing one of the four functional groups present in Prottremin (two double bonds and two hydroxyl groups) led to the disappearance or weakening of antiparkinsonian activity [12]. At the same time, it turned out that the attachment of an alkyl or thioalkyl substituent at position 9 makes it possible to obtain compounds with high antiparkinsonian activity [13].

Also, we determined monoepoxide of Prottremin (epoxydiol) as a biologically active metabolite and estimated its anti-PD activity in vitro and in vivo. In particular, our recent data indicate that epoxydiol supports cultured naïve and toxin-treated DA neurons, increases dopamine content in the brain, and alleviates motor symptoms of PD in experimental animals [14]. As epoxydiol does not bind to neurotrophic factors (NTFs) receptors in the cells and fails to elicit activation of standard prosurvival AKT downstream signaling cascade, it does not act via standard neurorestorative mechanisms activated, for example, by NTPs. At the same time, epoxydiol activated the ERK1/2 pathway, which is essential for the basal survival of Substantia nigra (SN) DA neurons [14]. In addition, the effective dose for epoxydiol was quite high compared to other neuroprotective agents belonging to small molecules.

Our previous experience and a better understanding of the structure-activity relationship of diol-like compounds allowed us to develop a library of compounds containing 20 Protremine derivatives and screen them on cultured primary DA neurons. Based on the screening results, we chose the most promising derivative (1*R*,2*R*,6*S*)-2-(1*H*-1,2,4-triazol-3-ylthio)-3-methyl-6-(prop-1-en-2-yl)cyclohex-3-enol (PA96) and tested its antiparkinsonian activity on two animal models: the toxin-based MPTP model and the haloperidol-induced catalepsy model.

Herein, we report the data on PA96 synthesis, its biological activity, acute toxicity, and pharmacokinetics. The PA96 showed biological activity in cultured DA neurons and two murine PD models. We showed PA96 to have an improved potency both in in vitro and in vivo assays. Indeed, PA96 effective concentration in survival assay for DA neurons compared to the related compound (epoxydiol) was 1−10 nM vs. 100 nM, and antiparkinsonian activity in vivo was 1 mg/kg for PA96 vs. 20 mg/kg for epoxydiol, accordingly. In addition, PA96 was found to penetrate BBB readily.

## 2. Results

### 2.1. Synthesis of PA96

**Figure 1:**
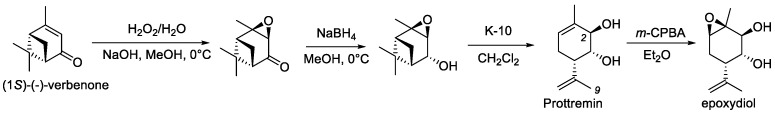
Synthesis of (1*R*,2*R*,6*S*)-3-methyl-6-(prop-1-en-2-yl)cyclohex-3-ene-1,2-diol (Prottremin).

Prottremin can be obtained in three stages from commercially available (1*S*)-(-)-verbenone in accordance with [9,11] (Figure 1).

**Figure 2:**
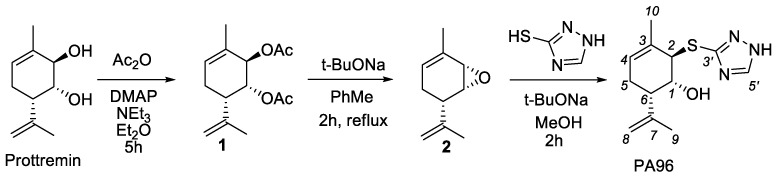
Synthesis of (1*R*,2*R*,6*S*)-2-(1*H*-1,2,4-triazol-3-ylthio)-3-methyl-6-(prop-1-en-2- yl)cyclohex-3-enol (PA96).

In this work, we for the first time carried out the synthesis (1*R*,2*R*,6*S*)-2-(1*H*-1,2,4-triazol-3-ylthio)-3-methyl-6-(prop-1-en-2-yl)cyclohex-3-enol (PA96). We have developed a unique procedure for the stereoselective synthesis of PA96 with the same stereochemistry of all asymmetric centers as in Prottremin (Figure 2), which will be described in detail elsewhere. The synthesis began with the preparation of acetate **1** according to the procedure [15], which was further converted into epoxide **2**. The reaction of epoxide **2** with 1*H*-1,2,4-triazole-3-thiol without additional purification yielded the target PA96.

### 2.2. PA96 Supports the Survival of Naïve and MPP^+^-Treated Dopamine Neurons

**Figure 3:**
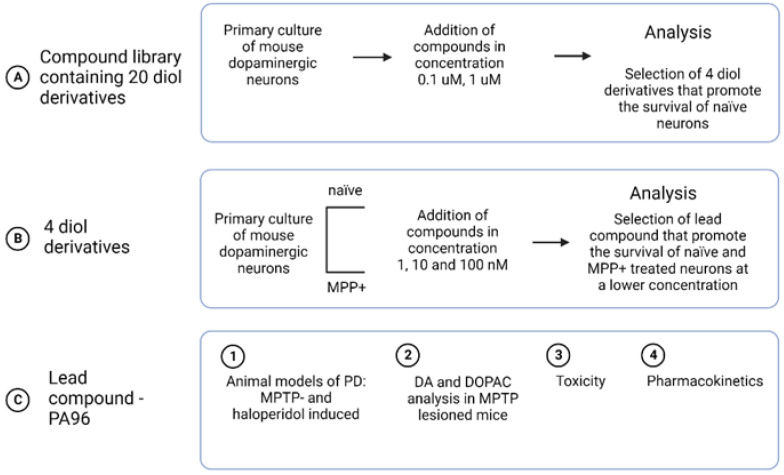
Scheme of the screening process. DA, dopamine; DOPAC, 3,4-dihydroxyphenylacetic acid; MPP^+^, 1-methyl-4-phenylpyridinium; MPTP, (1-methyl-4-phenyl-1,2,3,6-tetrahydropyridine); PD, Parkinson’s disease.

We developed a compound library containing 20 diol derivatives and screened them on cultured primary DA neurons (Supplementary Materials, Table S1). Compounds were taken at a dose of 0.1 and 1 μM (based on effective doses for the epoxydiol [14]). Based on the screening results, we selected four compounds and tested them in a naïve and MPP^+^-treated (1-methyl-4-phenylpyridinium (MPP^+^), the neurotoxic metabolite of MPTP) DA culture at 1, 10, and 100 nM to select the compound with the lowest effective dose. Finally, we chose one diol derivative, PA96, for further experiments (the scheme of the screening is shown in Figure 3).

**Figure 4:**
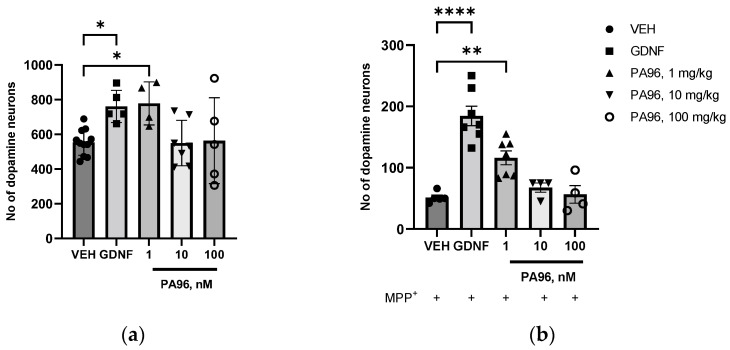
PA96, similarly to GDNF, promotes the survival of naïve (**a**) and MPP^+^-treated (**b**) primary midbrain dopamine neurons from wild-type mice in vitro. * *p* < 0.05, ** *p* < 0.01, **** *p* < 0.0001, ANOVA with Dunnett’s post hoc test (**a**) and paired *t*-test (**b**) compared to VEH. Each independent experiment is marked with a different shape by group: ●—VEH, ■—GDNF, ▲—PA96, 1 mg/kg, ▼—PA96, 10 mg/kg, ○—PA96, 100 mg/kg accordingly. The number of independent experiments (N) = 4–10. GDNF, glial cell line-derived neurotrophic factor; MPP^+^, 1-methyl-4-phenylpyridinium; VEH, vehicle.

PA96 promoted the survival of naïve tyrosine hydroxylase (TH)-positive neurons in a dose-dependent manner. In the wells treated with 1 nM PA96, the number of TH-positive neurons was 40% greater (*p* = 0.04, ANOVA with Dunnett’s post hoc test) than that in the vehicle (VEH)-treated wells (Figure 4a). PA96 protected cultured dopamine neurons against MPP^+^-induced degeneration. The number of TH-positive neurons in the wells treated with MPP^+^ and 1 nM PA96 was 2.4-fold greater than in VEH-treated wells (*p* = 0.03, paired *t*-test) (Figure 4b).

Finally, we chose one Prottremin derivative, PA96, and tested its antiparkinsonian activity in two animal models: a toxin-based MPTP model and a haloperidol-induced catalepsy model.

### 2.3. Antiparkinsonian Activity of PA96 In Vivo in Two Models of PD

The fact that the etiopathogenesis of PD is still unknown is the main obstacle preventing accurate PD modeling. Specific toxins, mechanical damage, genetic manipulation, and preformed α-synuclein fibrils are used to model PD in experimental animals [16,17,18]. Here, we used two animal models: the toxin-based MPTP model and the haloperidol-induced catalepsy model. Both models, dopamine depletion by MPTP or blockade of DA receptors by haloperidol, cause an imbalance in monoaminergic metabolism and are useful in drug screening for PD.

#### 2.3.1. MPTP-Induced Model

MPTP is the accepted and widely utilized neurotoxin for PD modeling because it recapitulates almost all the motor deficits and non-motor symptoms of PD and is very specific for the DA neurons of SN [19]. Indeed, due to MPTP-specific toxicity to DA neurons, it is used to test the status of the dopamine system (e.g., immunohistochemically using antibodies to markers of DA neurons in SN, such as TH, a key enzyme for DA synthesis, the dopamine transporter (DAT), the vesicular monoamine transporter 2 (VMAT2) or neuronal dyes such as Cresyl violet).

There are many variants of the MPTP protocol that differ in the mode of administration and dose of MPTP. In this study, we used the classic acute model with four injections of MPTP per day described previously [20].

However, for acute and even subchronic protocols, the DA neurons disappear rapidly. Their loss of TH immunoreactivity may not reflect actual cell death due to MPTP dramatically blocking TH gene expression and the TH-immunoreactive neurons beginning to “reappear” a few days after toxin administration [21]. In order to obtain more accurate estimates of DA neuronal loss in the acute model, we added one more injection of MPTP on the seventh day after the last one.

**Figure 5:**
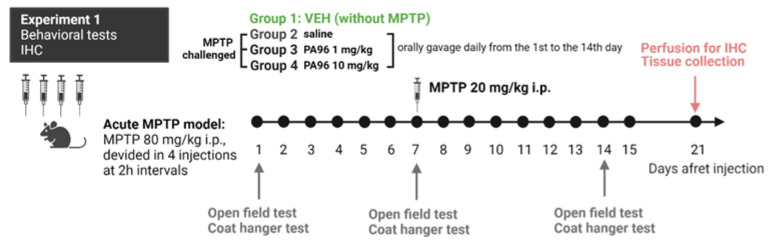
Study timeline of chronic experiment and the timing of assays. MPTP was injected intraperitoneally to C57Bl/6 male mice every 2 h in 8 h period on day 0 in a dose of 20 mg/kg for a total of four doses, and one additional dose of MPTP was injected on the seventh day. The timeline shows the treatment period of 14 days with PA96. IHC, immunohistochemistry; MPTP, 1-methyl-4-phenyl-1,2,3,6-tetrahydropyridine; VEH, vehicle.

The study timeline of the acute MPTP-based mouse PD model and the timing of behavioral assays are shown in Figure 5.

- Behavioral tests (open field, coat hanger)

To assess whether PA96 affects spontaneous locomotor activity, exploration, or anxiety-like behavior, we conducted the open field test 1 h after the first dose of PA96 and then every 7 days for 2 weeks.

**Figure 6:**
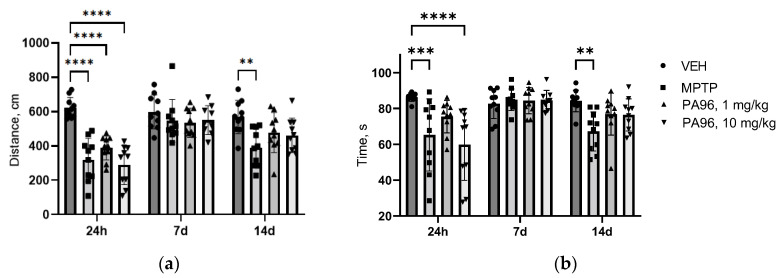
The influence of PA96 on locomotor activity of MPTP-treated mice: movement distance (**a**) and duration of locomotor activity (**b**) at 24 h, 7 d and 14 d after parkinsonism induction, respectively. Each column represents the experimental group, and independent experiment is marked with a different shape by group: ●—VEH, ■—MPTP, ▲—PA96, 1 mg/kg, ▼—PA96, 10 mg/kg accordingly. Mean ± SEM. N = 8–10 mice per group. ** *p* < 0.01, *** *p* < 0.001, **** *p* < 0.0001 compared to the VEH group, ANOVA with Dunnett’s post hoc test. MPTP, (1-methyl-4-phenyl-1,2,3,6-tetrahydropyridine); VEH, vehicle.

MPTP reduced the locomotor activity of mice, in particular, the movement distance and duration of locomotor activity at 24 h, 7 days, and 14 days after the last MPTP injection (Figure 6a,b). Administration of PA96 did not influence the movement distance in both doses, but 1 mg/kg brings duration of locomotor activity in animals at 24 h time point of experiment on VEH level (*p* = 0.1328 PA96 1 mg/kg vs. VEH). After 7 days, there were no differences between the groups. After 14 days in both doses, there was no significance between VEH and PA96.

**Figure 7:**
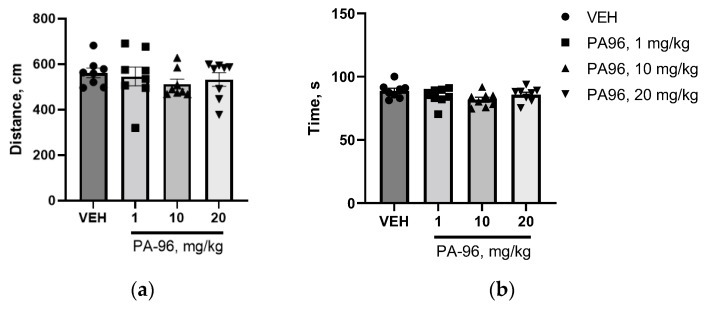
PA96 does not activate the locomotor activity of naïve mice: movement distance (**a**) and duration of locomotor activity (**b**). Each column represents the experimental group, and independent experiment is marked with a different shape by group: ●—VEH, ■—PA96, 1 mg/kg, ▲—PA96, 10 mg/kg, ▼—PA96, 20 mg/kg, accordingly. Mean ± SEM. N = 8–10 mice per group. There are no differences compared to the VEH group, ANOVA with Dunnett’s post hoc test. VEH, vehicle.

Moreover, in order to exclude psycho stimulation activity of PA96, a separate open field test on naïve male C57Bl/6 mice was conducted. PA96 did not activate the locomotor activity of naive animals (Figure 7a,b).

**Figure 8:**
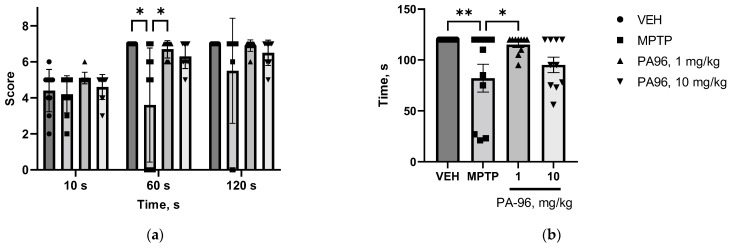
The influence of PA96 on motor coordination of MPTP-treated mice: appraised scores based on scoring criteria (**a**) and duration of movement activity (**b**). Each column represents the experimental group, and independent experiment is marked with a different shape by group: ●—VEH, ■—MPTP, ▲—PA96, 1 mg/kg, ▼—PA96, 10 mg/kg, accordingly. Mean ± SEM. N = 10 mice per group. * *p* < 0.05, ** *p* < 0.01 compared to the MPTP group, ANOVA with Dunnett’s post hoc test. MPTP, (1-methyl-4-phenyl-1,2,3,6-tetrahydropyridine); VEH, vehicle.

Next, the behavior was assessed using the coat hanger test. The coat hanger test was performed straight after the “open field” test on the same day (Figure 5). The VEH-treated mice reached the end of the bar more quickly compared to MPTP-treated animals. The coat hanger score at 60 s time point for VEH was 7.0, which means that 10 out of 10 mice reached the top of the coat hanger. At the same time, only two mice from the MPTP-treated group managed to climb the top of the coat hanger in 60 s, with the average score for this group being equal to 3.6 ± 1.0 (*p* = 0.0202) (Figure 8a). Mice treated with PA96 at a dose of 1 mg/kg showed significantly improved scores compared to MPTP (6.7 ± 0.2 vs. 3.6 ± 1.0, respectively, *p* = 0.0324) and the mice treated with the tested compound at the dose of 10 mg/kg showed a trend toward increasing the score in this test (*p* = 0.0622).

In addition, there were four falls at the 60 s and two falls at the 120 s time points in the MPTP group. All the animals from both vehicle- and PA-treated groups managed to stay on the coat hanger for the whole test duration (120 s). In other words, there were no falls in any other group except MPTP.

MPTP severely affected the motor activity of experimental animals by reducing the total time of active movements and increasing the immobility time. Indeed, during the trial, vehicle-treated mice were 46% more active than MPTP-treated animals. Administration of 1 mg/kg of PA96 to MPTP-treated mice increased the movement time by 40%, making this parameter comparable to that of healthy animals (Figure 8b).

- Evaluation of the neuroprotective properties of PA96 in the nigrostriatal dopamine system

Given the in vitro data showing the positive influence of PA96 on the survival of cultured DA neurons and a positive effect on behavioral tests, we decided to evaluate the neurorestorative properties of the tested compound in the nigrostriatal dopamine system. Therefore, we quantified the density of TH-positive fibers in the striatum (ST) and the number of TH-positive cells in SN.

**Figure 9:**
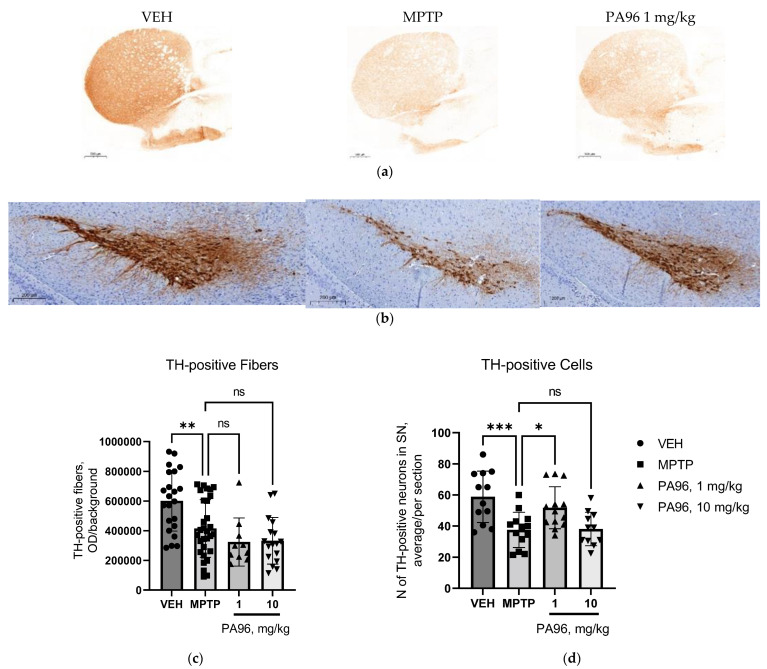
The influence of PA96 (1 and 10 mg/kg) on the density of TH-positive fibers in the striatum (**a**,**c**) and the number of TH-positive cells in SN (**b**,**d**) of MPTP-treated male C57Bl/6 mice. Each column represents the experimental group, and independent experiment is marked with a different shape by group: ●—VEH, ■—MPTP, ▲—PA96, 1 mg/kg, ▼—PA96, 10 mg/kg, accordingly. The data are presented as mean ± SEM, N = 3–6 per group. * *p* < 0.05, ** *p* < 0.01, *** *p* < 0.001 compared to the MPTP group, ANOVA with Dunnett’s post hoc test. MPTP, (1-methyl-4-phenyl-1,2,3,6-tetrahydropyridine); VEH, vehicle.

Administration of MPTP reduced the density of TH-positive fibers in the ST by 1.5 times compared to VEH-treated mice (Figure 9a). However, significant changes in the density of TH-positive fibers in the ST were not observed in any of the treatment groups.

We also studied the effect of PA96 on the number of TH-positive neurons in the SN. Treatment of MPTP mice with 1 mg/kg of PA96 increased by 37% the number of TH-positive neurons as compared to that in MPTP-treated mice (Figure 9)

#### 2.3.2. Antiparkinsonian Activity of PA96 in Haloperidol-Induced Catalepsy

Haloperidol-induced catalepsy is often used as a rodent model for studying motor deficits observed in PD and screening potential antiparkinsonian compounds. In contrast to MPTP, haloperidol does not cause neurodegeneration [20]. Haloperidol can induce extrapyramidal effects (mainly pseudo-parkinsonian symptoms such as catalepsy) by blocking postsynaptic D2 dopaminergic receptors in the mesolimbic system [22]. Catalepsy is a behavioral state of bradykinesia and rigidity in which the animal cannot correct externally imposed postures. Moreover, haloperidol causes mitochondrial dysfunction by inhibiting mitochondrial complex I and induces oxidative stress [23].

**Figure 10:**
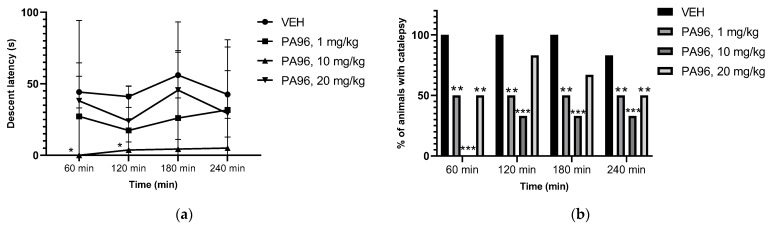
Time course (**a**) and % of animals with catalepsy (**b**) of parallel bars test showing the effect of PA96 on haloperidol-induced catalepsy. One-way ANOVA with Dunnett’s post hoc test analysis was used to compare descent latency between VEH and other groups *p* < 0.05 * vs. VEH. Chi-square analysis was used to compare the percent of the cataleptic response to haloperidol between mice with VEH and PA96-treated ** *p* < 0.01, *** *p* < 0.001 vs. VEH. VEH, vehicle.

PA96 significantly decreased catalepsy time (Figure 10a) and the percentage of cataleptic animals (Figure 10b).

Thus, compound PA96 demonstrated potent antiparkinsonian activity in the MPTP-induced PD model in mice and haloperidol-induced catalepsy in rats.

### 2.4. Effects of PA96 on Dopamine and Dopamine Metabolite-DOPAC Levels in MPTP-Lesioned C57BL/6 Mice

**Figure 11:**
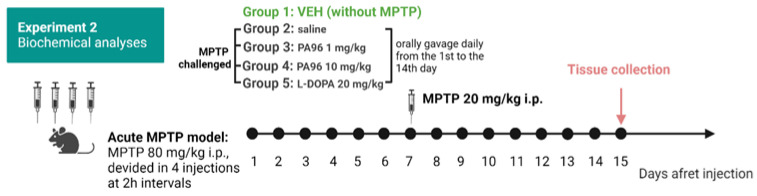
Study timeline of the experiment.

To further study the effect of PA96 on the dopaminergic system, we determined neurochemical profiles for DA and 3,4-dihydroxyphenylacetic acid (DOPAC) in the midbrain and ST (Figure 11).

**Figure 12:**
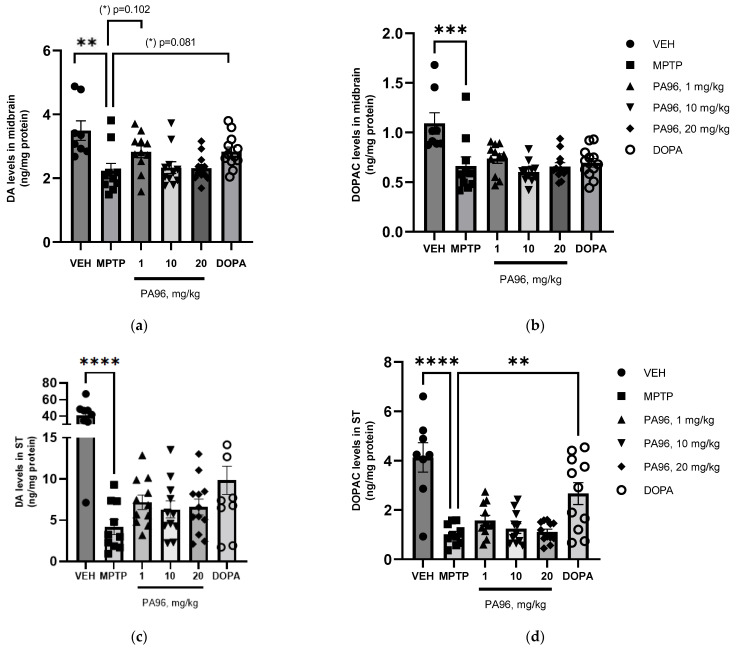
Effects of PA96 on DA and DOPAC levels after MPTP lesion. The concentrations of DADOPAC in the midbrain (**a**,**b**) and in the striatum (ST) (**c**,**d**) are presented as average ± SEM. Monoamine neurotransmitter metabolites are depicted in ng/mg of total brain protein. Each column represents the experimental group, and independent experiment is marked with a different shape by group: ●—VEH, ■—MPTP, ▲—PA96, 1 mg/kg, ▼—PA96, 10 mg/kg, ⯁—PA96, 20 mg/kg, ○—DOPA, accordingly. The statistical significances were analyzed by ANOVA with Dunnett’s post hoc test compared to MPTP. N = 10 mice per group. ** *p* < 0.01, *** *p* < 0.001, **** *p* < 0.0001. DA, dopamine; DOPAC, 3,4-dihydroxyphenylacetic acid; MPTP, (1-methyl-4-phenyl-1,2,3,6-tetrahydropyridine); ST, striatum; VEH, vehicle.

MPTP lesion significantly reduced the DA and DOPAC concentrations in both ST and midbrain (Figure 12). PA96 at a dose of 10 mg/kg did not affect the DA and DOPAC concentrations in either ST or midbrain. However, both PA96 and 1 mg/kg L-DOPA tended to alleviate the MPTP-induced reduction in DA level in the midbrain of experimental animals with comparable efficacy. In contrast to PA96, L-DOPA significantly increased the DOPAC level in ST.

### 2.5. Maximum Tolerated Dose and Toxicity of PA96 in Mice

First, we determined the maximum tolerated dose (MTD) of PA96 by employing CD-1 female mice. As shown in Table 1, 100% mortality was observed at 500 mg/kg. Abnormal clinical signs, such as piloerection, decreased motor activity, ptosis, and gait disturbance, were observed at 150 mg/kg and above. Thus, the established MTD for a single dose of PA96 was 100 mg/kg, which is 100-fold higher than the effective dose.

The LD_50_ value was determined by the arithmetic method of Kerber [24]. According to calculation, LD_50_ = 234 mg/kg was obtained.

### 2.6. Pharmacokinetics Study of PA96

When studying a new CNS-active compound, it is crucial to understand pharmacokinetic aspects, including a concentration–time profile in the brain, BBB transport, and brain accumulation, as well as the time of elimination from the brain. Successful penetration of the BBB is necessary for drugs to have central nervous effects. The brain-to-blood coefficient (*K_b,brain_*) is the most widely used in vivo parameter for evaluating the extent of CNS distribution. *K_b,brain_* is calculated as a ratio of the area under brain/blood pharmacokinetic curves between zero and 24 h (*K_b,brain_* = AUC*_brain_*/AUC*_blood_*). We should note here that this study aimed to estimate the content of new diol-based derivatives in blood and brain tissue samples from mice after the injection of a single oral dose. For more reliable data to be obtained, the developed method must be further validated according to the corresponding regulatory documents. Blood samples were collected from the tail after amputation of a small distal piece before mice were sacrificed to extract the brain samples. Then, the samples were prepared following the protocol (see Methods) and analyzed by high-performance liquid chromatography-tandem mass spectrometry (HPLC-MS/MS). Chromatographic conditions and mass spectrometer parameters for the analysis of PA96 and internal standards were optimized in a preliminary experiment.

**Figure 13:**
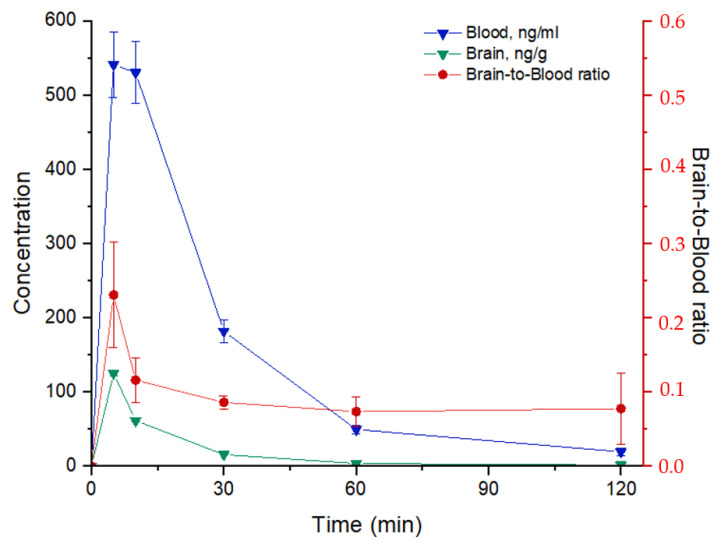
Concentration–time profile of PA96 in whole blood (blue curve) and brain tissue (green curve) samples; brain-to-blood concentration ratio-time profile of PA96 after single oral administration at the dose of 10 mg/kg (red curve). The data are presented as mean ± SEM, N = 3 per group.

The concentration–time curves of PA96 following its oral administration and selected pharmacokinetic parameters are shown in Figure 13 and Table 2, respectively. By summarizing the information about PA96 distribution in blood, it was found that the maximum concentration, C_max_ = (5.5 ± 0.5) × 10^2^ ng/mL, had started to drop 5 min after a single dosing and the minimum detectable concentration was observed after 1 h. Figure 12 shows that PA96 concentration in brain tissue samples rapidly reached a peak level at 5 min, C_max_ = (1.25 ± 0.04) × 10^2^ ng/g, demonstrating that PA96 penetrates into the brain rapidly without lag time. The concentration of the agent in the brain samples was close to the minimum value 2 h after administration. At 5 min, the concentration in the blood sample was approximately 4.5 times higher than the same value in the brain tissue sample. In addition, the main pharmacokinetic parameters, such as the area under the curve (*AUC*), mean residence time (*MRT*), and clearance (*CL*), were determined (Table 2). The blood-to-brain concentration ratio at each time point was calculated as the ratio of blood-to-brain concentration (*C*_blood_/*C*_brain_) (Figure 13). *K_b,brain_* was equal to 0.119 ± 0.010.

## 3. Discussion

Parkinson’s disease (PD) is an age-related neurodegenerative disease characterized by a severe loss of nigrostriatal dopamine neurons and dopamine depletion in the striatum, which causes typical motor symptoms, e.g., tremor, rigidity, and bradykinesia [2]. Currently, none of the available PD treatments has shown the ability to slow down the progression of neurodegeneration. Thus, there is an urgent medical need for a disease-modifying PD therapy. Brain-penetrant small molecules able to combat neurodegeneration are among the most prospective therapeutic modalities compared to biologicals tested in clinical trials [18,25,26]. Small molecules can be delivered systemically and spread in tissues well [5,6]. We have recently discovered a blood–brain barrier penetrating non-toxic molecule (1*R*,2*R*,6*S*)-3-methyl-6-(prop-1-en-2-yl)cyclohex-3-ene-1,2-diol (Prottremin) which is able to alleviate PD motor symptoms in toxin-based animal models of PD [8,9,10]. In addition, we determined Prottremin’s biological active metabolite epoxydiol, estimated its anti-PD activity in in vitro and in vivo, and obtained some insights into the molecular mechanism of its action [14].

In the present study, we evaluated the effects of the novel Prottremin derivative, a compound PA96, on dopamine neurons in vivo and in vitro, assessed its ability to alleviate motor deficits in animal models of PD, as well as PK profile and BBB penetration. PA96 was chosen from a library of Prottremin derivatives for its ability to support cultured dopamine neurons.

PA96 was synthesized from Prottremin using the original three-step stereoselective procedure. We found that PA96: (1) supported the survival of cultured näive dopamine neurons, (2) supported the survival of MPP^+^-challenged dopamine neurons in vitro and in vivo; (3) had chemically appropriate properties (synthesis, solubility, etc.), (4) alleviated motor deficits in MPTP- and haloperidol-based animal models of PD, (5) penetrated the blood–brain barrier in vivo, (6) was eliminated from the bloodstream relative rapidly.

Similarly to the related compound, epoxydiol, [14] PA96 supported the survival of cultured dopamine neurons in a dose-dependent manner. However, the potency of PA96 in this assay was significantly higher than that of the related compound: effective doses of epoxydiol were in the range of 0.1–1 µM while PA96 was effective already at 1 nM. Therefore, the potency of PA96 is up to 100 times higher than that of epoxydiol. Moreover, neither cytotoxic effect in naive neurons nor increased cytotoxicity with the administration of MPP^+^ were observed in in vitro experiments.

The dose-response curve of PA96 was not linear: we observed the superior efficacy of a lower PA96 dose (1 nM) compared to a larger one (10 and 100 nM). It is a common phenomenon in pharmacology and can be caused by several factors, including (1) toxicity of the higher doses of the studied compounds, (2) saturation and/or degradation of receptors, and (3) activation of negative feedback mechanisms in the cells in order to maintain homeostasis. To date, the only known substances able to support neuronal survival are neurotrophic factors (NTFs). NTFs bind to extracellular receptors and activate intracellular cascades necessary for neuronal survival, maintenance, and functioning. However, experimental evidence indicates that excessive and/or long-term cell stimulation with NTFs will not increase the neuronal capacity to survive but more likely lead to detrimental effects. It is related to the activation of negative feedback mechanisms and/or depletion of the receptor pool on the cell surface, as discussed in detail in [27]. Since PA96 also supports the survival of dopamine neurons, we assume that it also activates intracellular prosurvival cascades in the cells via specific yet-to-be-identified receptors. Thus, to explain this bell-shaped dose-response curve, we propose several hypotheses (1) PA96 may have off-target effects opposing their beneficial properties; (2) receptors activated by PA96 may become saturated, and therefore the response reaches a plateau or even decreases; and (3) negative feedback mechanisms might be activated in the cells. Obviously, further studies are needed to substantiate the underlying mechanisms.

Next, to evaluate the biological activity of PA96 in vivo, we used the MPTP-based animal model of PD, which allowed us to monitor both behavioral changes and the status of the dopamine system in experimental animals. To address motor deficits, we used two different tests: an open field to assess free movement and coat hanger tests to assess forced movement and motor coordination. We observed significant movement alterations induced by MPTP in both tests (open field and coat hanger) 24 h after the fourth toxin injection. However, these changes were short living and were not observed 7- and 14-days post MPTP. Indeed, the MPTP model of PD in the format used in this study comes with several limitations: (1) rodent locomotion is controlled in a different manner than that of humans, thus making the interpretation of rodent behavior as Parkinsonian following dopaminergic lesions very challenging [28,29]; (2) MPTP-treated mice shown a paucity of locomotion shortly after toxin administration, but these deficits disappeared over time, due to diminishing effects of the toxin on peripheral organs [30]; and (3) lack of specific deficits in motor behavior is not associated with any substantial loss of substantia nigra neurons or degeneration of striatal dopaminergic fibers [31]. Therefore, administration of PA96 (at a dose of 1 mg/kg) enhanced motor coordination in the coat hanger test and duration of activity in the open field test after 24 h, but not at the other studied time points. Altogether, these results show that PA96 in low doses improved overall motor deficits in MPTP-lesioned mice.

The results of our histological study demonstrate that MPTP significantly decreased the number of dopamine neurons and axonal fibers of the nigrostriatal pathway. PA96 in low concentration supported the survival of dopaminergic neurons in vivo. However, it is unclear whether PA96 can stimulate the outgrowth and arborization of neurites of dopamine neurons in the striatum since their loss induced by MPTP in this experiment was not alleviated by PA96. It is worth mentioning that neurodegeneration in PD starts from axons, and their regrowth takes time [32]. Therefore, PA96 promoted the survival of neuron cell bodies and might not have enough time for stimulation of neurites outgrowth. In addition, this data matched with those obtained for dopamine and its metabolite 3,4-dihydroxyphenylacetic acid (DOPAC) concentrations in the midbrain and striatum. MPTP-treated mice significantly reduced the dopamine and DOPAC concentrations in the striatum and midbrain. At the same time, PA96 tended to increase dopamine and DOPAC levels in the midbrain but not in the striatum.

In addition, to evaluate PA96 impact on the dopaminergic system, we used an animal model of haloperidol-induced catalepsy. PA96 significantly reduced the latency of descent from the bars in all tested doses and decreased % of cataleptic animals per group. This study confirms that PA96 can improve the behavioral alterations induced by non-selective dopaminergic receptor antagonist haloperidol. PA96 may exhibit this effect through several pathways: (1) alleviating dopamine depletion, (2) binding non-blocked dopaminergic receptors, and (3) activating the acetylcholinergic system. Moreover, according to our previous results, Prottremin demonstrated explicit activation of the cholinergic system [33]. Altogether, the proposed pathways may help eliminate imbalance of monoaminergic metabolism and mitigate the motor deficit.

Finally, the present study offers some insights into the pharmacokinetics of PA96. In mice, PA96 was shown to cross the blood–brain barrier rapidly, reaching a maximum concentration in the brain 5 min after iv administration. The elimination half-life of PA96 constituted approximately 24 min. Such rapid pharmacokinetics may have pros and cons regarding neurorestorative treatment development. On the one hand, rapid action may help overcome negative long-term stimulation of neurons and, as previously discussed, for NTFs. In addition, if PA96 has a similar mechanism as NTFs activating some intracellular cascades important for neuronal survival, short-term stimulation might be sufficient to initiate the process, which would further develop in the absence of stimulus to produce long-lasting effects. On the other hand, neuroprotective therapy could be considered a replacement therapy, which must constantly be in the brain. Thus, further research with prolonged forms of PA96 might be of interest to determine the best candidate for subsequent clinical trials.

Finally, we evaluated the acute toxicity of PA96 in mice. Our test results indicate that PA96 with LD_50_ < 300 belongs to Category III (slightly hazardous) [34]. However, the therapeutic dose for PA96 was at least 100 times lower than that for LD50, and both doses, 1 mg/kg and 10 mg/kg administered for 14 days, were well tolerated. Thus, PA96 was determined to have a high therapeutic index.

## 4. Methods

### 4.1. General Methods and Materials

All commercially available compounds and solvents were reagent grade and were used without further treatment unless otherwise noted. Column chromatography (CC): silica gel (SiO_2_; 60–200 μ; *Macherey-Nagel*); hexane/EtOAc 100:0 → 0:100. GC/MS (purity control and products analysis: Agilent 7890A (Agilent Technologies, Santa Clara, CA, USA)) with a quadrupole mass spectrometer Agilent 5975C as a detector, HP-5MS quartz column, 30,000 × 0.25 mm, He (1 atm) as carrier gas. Optical rotation: *polAAr 3005* spectrometer (Optical Activity LTD, Huntingdon, UK, CHCl_3_ soln. HR-MS: *DFS-Thermo-Scientific* spectrometer in a full scan mode (15–500 *m*/*z*, 70 eV electron-impact ionization, direct sample introduction) (Thermo Fisher Scientific, Waltham, MA, USA and Agilent 7200 Accurate Mass Q-TOF GC/MS (70 eV, electron-impact ionization (Agilent Technologies, Santa Clara, CA, USA). ^1^H- and ^13^C-NMR: *Bruker* Avance-III 600 (Bruker Corporation, Karlsruhe, Germany) apparatus at 600.30 MHz (^1^H) and 150.95 MHz (^13^C) and *Bruker* Avance 400 (Bruker Corporation, Karlsruhe, Germany) apparatus at 400.13 MHz (^1^H) and 100.61 MHz (^13^C) in CDCl_3_; chemical shifts d in ppm rel. to residual CHCl_3_ (d(H) 7.24, d (C) 76.90 ppm), *J* in Hz. Structure determinations: by analyzing the ^1^H NMR spectra, including ^1^H-^1^H double resonance spectra and ^1^H-^1^H 2D homonuclear correlation (COSY, NOESY); J-modulated ^13^C NMR spectra (JMOD), and ^13^C-^1^H 2D heteronuclear correlation with one-bond and long-range spin-spin coupling constants (C-H COSY, ^1^*J*(C,H) = 135 Hz; HSQC, ^1^*J*(C,H) = 145 Hz; HMBC, ^2,3^*J*(C,H) = 7 Hz). All the target compounds reported in this paper have a purity of at least 95%. (1*R*,2*R*,6*S*)-3-Methyl-6-(prop-1-en-2-yl)cyclohex-3-ene-1,2-diol was synthesized according to [2] from (1*S*)-(-)-verbenone (*Aldrich*). (1*R*,2*R*,6*S*)-3-Methyl-6-(prop-1-en-2-yl)cyclohex-3-ene-1,2-diacetate was synthesized according to [11].

### 4.2. Synthesis of (1R,2R,6S)-2-(1H-1,2,4-triazol-3-ylthio)-3-methyl-6-(prop-1-en-2-yl)cyclohex-3-enol PA96

The mixture of sodium *tert*-butoxide (800.7 mg, 8.33 mmol) and 10 mL toluene was warmed to the boiling point of the solvent in a glycerin bath. After, (1*R*,2*R*,6*S*)-3-methyl-6-(prop-1-en-2-yl)cyclohex-3-ene-1,2-diacetate **1** (525.6 mg, 2.08 mmol) dissolved in 5 mL toluene was added dropwise to the reaction mixture. The mixture was stirred at reflux at 110 °C for 2 h. Afterward, the reaction mixture was cooled down, 30 mL hexane was added. After the end of precipitation, the solution is filtered from the precipitate and evaporated *in vacuo*. This yielded (1*S*,5*S*,6*R*)-2-methyl-5-(prop-1-en-2-yl)-7-oxabicyclo [4.1.0] hept-2-ene **2**.

Yellow oil. Yield = 75%. ${\left[\alpha \right]}_{D}^{25.3}$ = 1.96(c = 0.46, CHCl_3_). ^1^H-NMR (600 MHz, CDCl_3_, δ, ppm): 1.81 (3H, s, CH_3_-9), 1.86–1.87 (3H, m, CH_3_-10), 1.88–1.99 (2H, m, CH_2_-5), 2.30–2.35 (1H, m, CH-6), 3.09 (1H, dd, J(2,1) = 4.4 Hz, J(2,4) = 2.3 Hz, CH-2), 3.4 (1H, d, J(1,2) = 4.4 Hz, CH-1), 4.82–4.85 (1H, m, CH_2_-9), 4.88–4.9 (1H, m, CH_2_-9), 5.6–5.64 (1H, m, CH-4). ^13^C-NMR (150 MHz, CDCl_3_, δ, ppm): 146.72 (C, C-7), 130.26 (C, C-3), 125.59 (CH, C-4), 111.22 (CH_2_, C-8), 58.27 (CH, C-1), 51.18 (CH, C-2), 40.71 (CH, C-6), 26.28 (CH_2_, C-5), 21.38 (CH_3_, C-10), 20.61 (CH_3_, C-9). HR-MS: 150.1038 (M^+^, C_10_H_14_O^+^; calc. 150.1039).

1*H*-1,2,4-Triazole-3-thiol (207.3 mg, 2.05 mmol) was added to sodium *tert*-butoxide (197 mg, 2.05 mmol) dissolved in 10 mL methanol. After 5 min solution of compound **2** (1.67 mmol) in CH_3_OH (10 mL) was added to the mixture when stirred. The reaction mixture was stirred for 2 h at r.t. Then, the solvent was distilled off, and the residue was extracted with ethyl acetate (3 × 10 mL). The organic phase was dried over Na_2_SO_4_, filtered, and evaporated in vacuo. The residue was purified by column chromatography on SiO_2_.

Colorless oil. Yield = 60%. ${\left[\alpha \right]}_{D}^{25.3}$ = −34.6(c = 0.67, CHCl_3_). ^1^H-NMR (400 MHz, CDCl_3_, δ, ppm): 1.72 (3H, s, CH_3_-9), 1.79 (3H, s, CH_3_-10), 2.0–2.09 (1H, m, CH_2_-5), 2.21–2.34 (1H, m, CH_2_-5), 2.60 (1H, dd, J(6a,5a) = 11.9 Hz, J(6a,5e) = 5.2 Hz, CH-6), 4.11 (1H, br.s, CH-2), 4.27 (1H, s, CH-1), 4.82 (1H, s, CH-8), 4.87 (1H, s, CH-8), 5.69 (1H, s, CH-4), 8.23 (1H, s, CH-5′). ^13^C-NMR (100 MHz, CDCl_3_, δ, ppm): 145.53 (C, C-3′), 145.48 (C, C-7), 127.22 (CH, C-5′), 127.18 (C, C-3), 111.25 (CH_2_, C-8), 70.57 (CH, C-1), 52.42 (CH, C-2), 38.80 (CH, C-6), 24.47 (CH_2_, C-5), 22.05 (CH_3_, C-9), 21.91 (CH_3_, C-10). HR-MS: 251.1085 (M^+^, C_12_H_17_O_1_N_3_^32^S_1_^+^; calc. 251.1087).

### 4.3. Survival of Naïve and MPP^+^-Challenged Wild-Type Dopamine Neurons

The survival of cultured mouse DA neurons was assessed as described before [14,35]. Briefly, naïve or MPP^+^ (dopaminergic selective neurotoxin)-challenged embryonic midbrain neurons were cultured in the presence or absence of a diol-like compound. The number of survived DA neurons was estimated after immunocytochemical staining with antibodies recognizing the key enzyme of dopamine synthesis—tyrosine hydroxylase [2].

### 4.4. Experimental Animals

Male C57BL/6 mice, female CD-1 mice, and male albino Wistar rats were obtained from the SPF vivarium of the Institute of Cytology and Genetics, Russia. The mice weighed 25–30 g, and the rats weighed 200–225 g at the beginning of the experiment.

The animals were housed in groups of eight to ten for mice and six for rats in clear plastic cages with a layer of sawdust in ambient temperature (22–24 °C) and artificial lighting with a fixed 12 h light-dark cycle. Pellet food and tap water were available *ad libitum*.

All animal experiments were carried out in accordance with the guidelines laid down in the legislation of the Russian Federation and European Communities Council Directive of 24 November 1986 (86/609/EEC) and were approved by the County Administrative Board of Southern Finland (license number: KEK15-022).

### 4.5. Antiparkinsonian Activity of PA96 In Vivo

#### 4.5.1. The MPTP Mouse Model of Parkinson’s Disease Induced by MPTP Neurotoxin

MPTP was injected intraperitoneally into mice of C57Bl/6 line every 2 h in 8 h period in one day in a dose of 20 mg/kg for a total of four doses as described before [20], and one additional dose of MPTP was injected on the 7th day. The studied compound PA96 was dissolved in saline containing 0.5% Tween 80 just before use. PA96 in a dose of 1 and 10 mg/kg or VEH was administrated per os 24 h after the last injection of MPTP. Then, PA96 or VEH was injected for 14 days in a 5-day-per-week regime for a total of fifteen doses.

#### 4.5.2. Behavioral Tests (Open Field and Coat Hunger Tests)

The effectiveness of the studied medications was evaluated according to their ability to reduce the symptoms of hypokinesia induced by MPTP on days 1, 7, and 14 of the experiment. Hypokinesia caused by neurotoxin administration was evaluated with the “open field” test performed for 2 min using Tru Scan (U.S.) 2 h after the administration of the studied compound, registering the main markers of the locomotor and exploratory activities: time of locomotor activity (s) and movement distance (cm).

Hypokinesia caused by neurotoxin administration was also evaluated with the coat hanger test. The coat hanger test is commonly used to evaluate forelimb strength and coordination. The test was performed as described previously by Mätlik et al. [36] with some modifications in the score. In short, the animal was placed with forepaws hanging a point halfway across of horizontal bar. The body position of the animal was observed for 120 s and was scored as follows:

0—Falls off within 10 s;

1—Hangs onto the bar with one forepaw;

2—Hangs onto the bar with two forepaws;

3—Hangs onto the bar with two forepaws plus one hind paw;

4—Hangs with all four paws;

5—Hangs by all four paws plus tail wrapped around the bar, attempts to climb onto the bar;

6—Active escape to the end of the bar and reaches the corner;

7—Reaches the top of the coat hunger.

The total time of movement and the moments of freezing within 120 s were also evaluated.

### 4.6. Assessment of Neurorestorative Properties of PA96 In Vivo

#### 4.6.1. Sample collection and Tissue Processing

The samples were collected 7 days after the last administration of PA96. Mice were anesthetized using terminal phenobarbital injection (50 mg/kg), perfused trans-cardially with PBS, and then 4% paraformaldehyde in PBS in accordance with [37]. The brains were collected, treated, and embedded into paraffin blocks using the histological complex Microm (Fürth, Germany).

#### 4.6.2. Immunohistochemistry

To analyze the effect of PA96 in dopamine neurons in vivo, tyrosine hydroxylase immunohistochemistry was performed as described previously [38]. The PFA-fixed and paraffin-embedded brains were cut into 5 µm thick sections (3 sections per slide), and every fifth slide was taken for TH staining. The sections were deparaffinized, followed by a citrate antigen retrieval procedure. Endogenous peroxidase was inactivated by 30 min incubation with 3% H_2_O_2_. The sections were blocked with 5% normal goat serum and then probed with monoclonal TH antibody (1:2000, Millipore Cat# MAB318 Lot# RRID:AB_2201528) overnight at +4 °C. The sections were washed, and biotinylated horse antimouse secondary antibody (1:200, Cat# BA-2001, Vector Laboratories, Burlingame, CA, USA) was applied for 1 h, followed by washing. The sections were treated with ABC and DAB staining kits (Vector Laboratories, CA, USA) according to the manufacturer’s instructions to visualize bound antibodies.

#### 4.6.3. Optical Density and TH-Positive Neurons

The sections were scanned with an automated scanner (3DHistech, Budapest, Hungary), and the images were converted to 16-bit grayscale. The signal from cortical staining was used to measure nonspecific background staining. The integrated optical densities in images were measured in ImageJ (NIH) and divided by area in pixels.

### 4.7. Antiparkinsonian Activity of PA96 in Haloperidol-Induced Catalepsy

Neuroleptic-induced catalepsy has long been used as an animal model for screening drugs for parkinsonism, as described before [10,22]. Adult male Wistar rats were divided into four groups, each containing six animals. Group 1 received haloperidol 1.5 mg/kg in normal saline and served as the cataleptic control without any drug treatment. The studied compound PA96 was dissolved in saline containing 0.5% Tween 80 just before use. Groups 2, 3, and 4 received PA-96 in a dose of 1, 10, or 20 mg/kg per os accordingly; 10 min after the PA96 administration, catalepsy was induced by the intraperitoneal administration of haloperidol in a dose of 1.5 mg/kg body weight in normal saline. All the behavioral studies were performed at room temperature in a calm room without external interference. The severity of catalepsy was measured 30, 60, 120, 180, and 240 min after haloperidol administration using the method of parallel bars. The fore and hind limbs of a rat were placed on parallel bars (walls) to ensure that the animal’s back was straight, and the time spent by the animal in an immobilized state was recorded. The general duration of catalepsy and the percentage of cataleptic animals in the group were evaluated.

### 4.8. Neurochemical Analyses via HPLC-ED

Neurochemical analyses via HPLC-ED were conducted as described before [39]. Microdissected samples of the striatum (ST) and midbrain were homogenized in 300 and 400 µL of cold 0.6 M HClO_4_, respectively, using a motor-driven grinder (Z359971, Sigma-Aldrich, USA) and the homogenate was spun for 15 min at 12,000 rpm (+4 °C). The pellet was diluted in 1 mL of 0.1 M NaCl and used for protein quantitation by Bradford (Bio Rad, USA) according to the manufacturer’s protocol. The clear supernatant was diluted twice with pure water and used for assay of DA and 3,4-hydroxyphenylacetic acid (DOPAC) by HPLC on Luna C18(2) column (5 μm particle size, L × I.D. 75 × 4.6 mm, Phenomenex, Torrance, CA, USA) with electrochemical detection (750 mV, DECADE II™ Electrochemical Detector; Antec, The Netherlands). The standard mixes containing 1, 2, and 3 ng of DA and DOPAC were repeatedly assayed throughout the entire procedure and used to plot the calibration curves for each substance. The areas of peaks were estimated using LabSolution LG/GC software version 5.54 (Shimadzu Corporation, Japan) and calibrated against the calibrated curves for corresponding standards. The contents of DA and DOPAC were expressed nanogram (ng) per milligram (mg) of protein assayed by Bradford.

### 4.9. Influence of PA96 on Naïve C57bl/6 Mice

The studied agent in a dose of 1 mg/kg, 10 mg/kg, and 20 mg/kg or saline was administered once *per os* in a water-Tween 80 solution to 8 mice per group of C57Bl/6 mice. The main markers of the locomotor activity movement distance (cm) and duration of locomotor activity (s) were registered with the open field test performed for 2 min using Tru Scan (USA), 1 h after the administration of the studied agent.

### 4.10. Maximum Tolerated Dose and LD50 of PA96 In Vivo

Healthy animals were randomly divided into groups of four males or four females each. After a 3–4 h starvation, animals were administered 100, 150, 200, or 500 mg/kg PA96 solution (prepared by dissolution in saline containing 0.5% Tween 80) or the vehicle (sterile distilled water). Food was provided for approximately 1–2 h after administration. All mice were continuously monitored for changes in weight, breathing, ptosis, gait disturbance, tremor, and mortality 1 h after administration of PA96 and every 6 h after that. The day of dosing was designated as day 0, and animals were observed until day 14. Each mouse was weighed on days 0, 1, 3, 7, and 14.

The arithmetic method of Kerber was used to determine the lethal dose fifty (LD50) that killed 50% of studied animals with PA96.

The LD_50_ value was determined according to the following equation [24]:

LD_50_ = LD_100_ − ∑ (z × d)/m (1)

where LD_50_—dose capable of killing 50% of animals; LD_100_—dose capable of killing 100% of animals; z—half the sum of the number of animals in the experiments with the test of two following doses; d—the difference between two successive doses of administrative PA96; m—total number of animals in each group.

### 4.11. Pharmacokinetics of PA96

#### 4.11.1. Pharmacokinetic Study of PA96 in Whole Mouse Blood

Six CD1 IGS mice were administered orally with the compound PA96 in Tween 80 at doses of 10 mg/kg. Blood was collected from the tail after the amputation of a small distal piece. Time points were 5, 10, 30, 60, and 120 min after injection. Experimental blood samples were transferred into tubes for the precipitation of blood proteins with a mixture of zinc sulfate and methanol (2:8, *v*/*v*). A set of calibrators were prepared in advance, followed by their preparation protocol, and analyzed by HPLC-MS/MS for standard comparison. The calibration curve was built in the range of 25–2000 ng/mL. According to the signal-concentration profile, the content of PA96 in the experimental blood samples at each time point was calculated.

- Chemicals and Reagents

2-Adamantylamine hydrochloride used as an IS was purchased from Sigma-Aldrich (Burlington, USA). The homogenization mixture VetexQ Tox for QuEChERS was purchased from Interlab (Moscow, Russia). Analytical-grade methanol was purchased from Merck (Darmstadt, Germany), and zero-grade acetonitrile was purchased from Cryochrom (Saint-Petersburg, Russia). Formic acid was purchased from Panreac (Barcelona, Spain). High-purity water was prepared using a Direct-Q 3 UV system (Millipore S. A. S., France).

- Preparing solutions

The stock solution of PA96 (1.0 mg/mL) was prepared by dissolving an accurately weighed amount of the substance in 100% methanol. To obtain a set of working solutions with concentrations of 0.01, 0.05, 0.1, 0.25, 0.5, 1, 2.5, 5, 7.5, 10, 15, and 20 µg/mL, the stock solution of PA96 was diluted with methanol. Calibration standards (calibrators) were prepared by mixing 10 μL of each individual working solution and 90 μL of mouse whole blood. Samples were stirred gently on an orbital shaker at 1000 rpm and left at 4 °C for 1 h for equilibration. The stock solution of IS with a concentration of 1.0 mg/mL was prepared by dissolving a weighed amount of 2-adamantylamine hydrochloride in pure methanol. The solution for the protein precipitation procedure was a mixture of 2 parts of 0.2 M aqueous solutions of zinc sulfate per 8 parts of a solution of IS in methanol with a concentration of 10 µg/mL. All solutions were stored at −18 °C and brought to their ambient temperature before use.

- Blood Sample Preparation protocol

An aliquot of 10 µL of each whole blood sample was placed into an Eppendorf microtube (1.5 mL), then the precipitation solution of 50 µL was added to each tube. All samples were vortexed for 20–30 s and then centrifuged (Eppendorf MiniSpin, Eppendorf, Germany) for 10 min at 13,400 rpm. The supernatant was transferred into a chromatographic vial and analyzed.

#### 4.11.2. Pharmacokinetic Study of PA96 in Brain Tissue Samples

CD1 IGS mice received a single oral dose of PA96 in Tween 80 (10 mg/kg). Mice were sacrificed 5, 10, 30, 60, and 120 min after injection, and all brain samples were collected and placed into an Eppendorf microtube (2 mL). Samples were frozen with liquid nitrogen for subsequent preparation and HPLC-MS/MS analysis. A set of calibrators were prepared, followed by their preparation protocol, and analyzed. The calibration curve was built in the interval of 1–100 ng/g and used to calculate the PA96 concentration in the brain tissue samples.

- Chemicals and Reagents

2-Adamantylamine hydrochloride used as an IS was purchased from Sigma-Aldrich (Burlington, USA). The homogenization mixture VetexQ Tox for QuEChERS was purchased from Interlab (Moscow, Russia). Analytical-grade methanol was purchased from Merck (Darmstadt, Germany), and zero-grade acetonitrile was purchased from Cryochrom (Saint-Petersburg, Russia). Formic acid was purchased from Panreac (Spain). High-purity water was prepared using a Direct-Q 3 UV system (Millipore S. A. S., France).

- Preparing solutions

A stock solution was prepared by dissolving approximately 10 mg of PA96 in a required amount of pure methanol to have the solution with a concentration of 1.0 mg/mL. The working solutions of PA96 with concentrations of 5, 7.5, 25, 50, 75, 250, and 300 ng/mL were prepared by diluting the stock solution with methanol. To prepare a calibrator, a free PA96 brain tissue (~50 mg) was weighed and transferred into an Eppendorf microtube (V = 1.5 mL), then the working solution of PA-96 was added. The mixture was homogenized (Minilys, Bertin Technologies, Montigny-leBretonneux, France) for 60 s at low speed. The calibrators were prepared on the day of the experiment. The working solution of IS in acetonitrile with a concentration of 1.0 mg/mL was used. All the solutions were stored at −18 °C. Before sample preparation, the solutions were brought to room temperature.

- Brain Tissue Homogenization Protocol

The exact amount (~50 mg) of the mouse brain tissue sample was transferred into a polypropylene tube (1.5 mL). For each portion, the solution of IS (1 µL per 1 mg of the brain sample) and pure acetonitrile (10 µL per 1 mg of the brain sample) were added, then all samples were homogenized for 4 min at medium speed. After the homogenization procedure, ~100 mg of the salt mixture (magnesium sulfate: sodium chloride: trisodium citrate: disodium citrate as 4:1:1:0.5) for extraction was added, followed by shaking for 30 min at 1400 rpm in an orbital shaker. The supernatant was separated from the homogenate by centrifugation for 10 min at 13,400 rpm. The obtained solution was further shaken with ~80 mg of the SPE mixture (magnesium sulfate:C18EC as 6:1 and acetonitrile) for 10 min at 1400 rpm and centrifuged for 10 min at 13,400 rpm. A total of 300 µL of the organic phase was placed into a fresh tube and evaporated to dryness in a vacuum. The residue containing the analyte and IS was dissolved in 150 μL of methanol, shaken for 10 min, transferred into a chromatographic vial, and analyzed by HPLC-MS/MS.

#### 4.11.3. HPLC-MS/MS Conditions for Determining PA96

The LC-MS/MS analysis was performed using an LC-20AD Prominence chromatograph equipped with an autosampler thermostated at +10 °C (Shimadzu, Japan) and a 3200 QTRAP mass spectrometer equipped with an ESI source (AB SCIEX, USA). Separation was achieved on a ProntoSil-120-5-C18 AQ column (2.0 × 75 mm, 5 μm particles, (EcoNova, Novosibirsk, Russia)) thermostated at +35 °C. The mobile phase was composed of water containing 0.1% formic acid (eluent A) and methanol containing 0.1% formic acid (eluent B). The gradient elution program was as follows: 0 min-10% (B); 0.5 min-10% (B); 7 min-98% (B); 9 min-98% (B); the flow rate was 200 μL/min.

The mass spectrometer was operated using multiple reaction monitoring (MRM) in positive ion mode. Ionization was conducted by applying a voltage of 5500 V, and the source temperature was set at +350 °C. For the analyte and IS, the optimized source parameters, viz. CUR (curtain gas), GS1 (Gas 1), and GS2 (Gas 2) were set at 20 psi. The CAD (collisionally activated dissociation) gas was nitrogen, and the parameter was set as high. The parameters of PA96, viz. MRM transitions, declustering potentials (DP), collision energies (CE), and collision cell exit potentials (CXP) are presented in Table 3. The dwell time was 100 msec. Analyst 1.6.2 software (AB SCIEX, USA) was used for instrument control and data acquisition, and MultiQuant 2.1 software (AB SCIEX, USA) was used for quantification. To calculate the main pharmacokinetic parameters, the Microsoft Excel–PKSolver was used [40].

### 4.12. Statistical Analysis

Statistical analysis of the data was performed using one-way ANOVA with a Dunnett’s post hoc test or paired *t*-tests to compare multiple treatment groups, and frequency values were analyzed using Pearson’s chi-square test to determine the effect of PA96 on catalepsy manifestations in haloperidol-treated mice in Graphpad Prism 9 software (La Jolla, CA, USA). All data are presented as mean ± SEM. P-values below 0.05 were considered to indicate statistically significant differences between groups. No exclusions were made from the data from behavioral tests.

## 5. Conclusions

We have found that PA96 protects cultured dopamine neurons against spontaneous and toxin-induced death of DA neurons. In animals treated with MPTP, PA96 increases the density of TH-positive neurons and DA concentration in the midbrain. Moreover, the latter might be related to neurorestorative and/or DA metabolism changes.

Also, our data indicate that PA96 produces immediate positive effects in the haloperidol-induced PD model. Hence, PA96 may encourage the development of disease-modifying treatment against PD. The compounds developed have low toxicity and are characterized by rapid pharmacokinetics.

Taken together, our results suggest that PA96 may be promising both for treating disease progression in PD and alleviating PD symptoms. The mechanism of action for PA96 is yet to be determined.

## Figures and Tables

**Table 1:** Determination of maximum tolerated dose of PA96 administered orally.

Dose Level (mg/kg/day)	Mortality, %
500	100
200	60
150	50
100	0

**Table 2:** Pharmacokinetic parameters of PA96 in blood and brain tissue samples after single oral administration at the dose of 10 mg/kg.

Parameter	Blood	Units	Brain	Units
t_1/2_	22.8 ± 1.5	min	18.8 ± 0.3	min
T_max_	5	min	5	min
C_max_	(5.5 ± 0.5) × 10^2^	ng/mL	(1.25 ± 0.04) × 10^2^	ng/g
AUC_0-t_	(16.7 ± 1.5) × 10^3^	ng/mL∗min	(1.99 ± 0.09) × 10^3^	ng/g∗min
AUC_0-inf_obs_	(17.4 ± 1.8) × 10^3^	ng/mL∗min	(2.03 ± 0.09) × 10^3^	ng/g∗min
MRT_0-inf_obs_	29.7 ± 2.2	min	21.5 ± 0.7	min
Vz/F__obs_	(18.9 ± 0.6) × 10^−3^	(ml/kg)/(ng/mL)	(133 ± 4) × 10^−3^	(mg/kg)/(ng/g)
Cl/F__obs_	(5.8 ± 0.6) × 10^−4^	(mg/kg)/(ng/mL)/min	(50 ± 1) × 10^−4^	(mg/kg)/(ng/g)/min

**Table 3:** Multiple reaction monitoring parameters for the analyte and IS.

Analyte and Precursor Ion (*m*/*z*)	Product Ion (*m*/*z*)	DP (V)	CE (V)	CXP (V)
PA96 252.2	102.1 (quantifier)	21	23	4
	133.2 (qualifier)	21	21	4
	151.3 (qualifier)	21	17	4
IS 152.3	93.1 (qualifier)	16	35	14
	107.2 (qualifier)	21	37	8

DP—declustering potential, CE—collision energy, CXP—collision cell exit potential.

## Data Availability

Data are not available.

## Supplementary Materials

The following supporting information can be downloaded at: https://www.mdpi.com/article/10.3390/molecules27238286/s1, ^1^H and ^13^C NMR spectra and HR-MS spectrum of PA-96 (Figures S1–S6); results of screening diol derivatives on naïve and MPP^+^-treated DA neurons (Table S1).

## Sample Availability

Samples of the compounds are available from the authors.
