# Supplementary material for: A Newly Identified Monoterpenoid-Based Small Molecule Able to Support the Survival of Primary Cultured Dopamine Neurons and Alleviate MPTP-Induced Toxicity In Vivo
Source: Molecules. 2022 Nov 28;27(23):8286. doi: 10.3390/molecules27238286 (PMC9738927; doi:10.3390/molecules27238286)
Supplement: Supplementary file 1 [file molecules-27-08286-s001.zip › molecules-2014324-supplementary.pdf]

Supplementary material

A newly discovered small molecule able to support the survival of primary cultured dopamine neurons and alleviate MPTP-induced toxicity *in vivo*

Anastasiia Kotliarova [1,2], Alexandra V. Podturkina [1], Alla V. Pavlova [1], Daria S. Gorina [1,3], Anastasiya V. Lastovka [1,3], Oleg V. Ardashov [1], Artem D. Rogachev [1,3], Arseniy E. Izyurov [4], Alla B. Arefieva [4], Alexander V. Kulikov [4], Tatyana G. Tolstikova [1], Konstantin P. Volcho [1,*], Nariman F. Salakhutdinov [1], Yulia Sidorova [2,*]

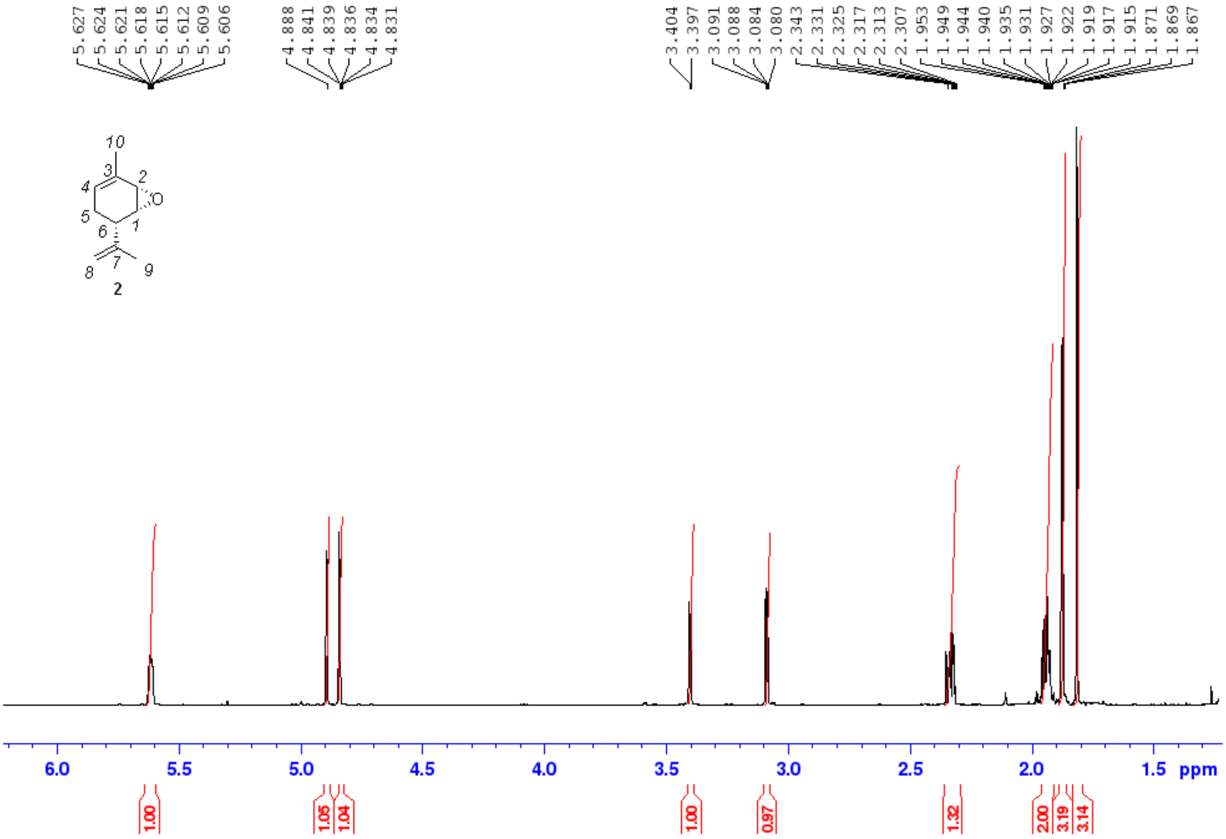

Figure S1: ^{1}H (600 MHz, $CDCl_3$) spectrum of **2**

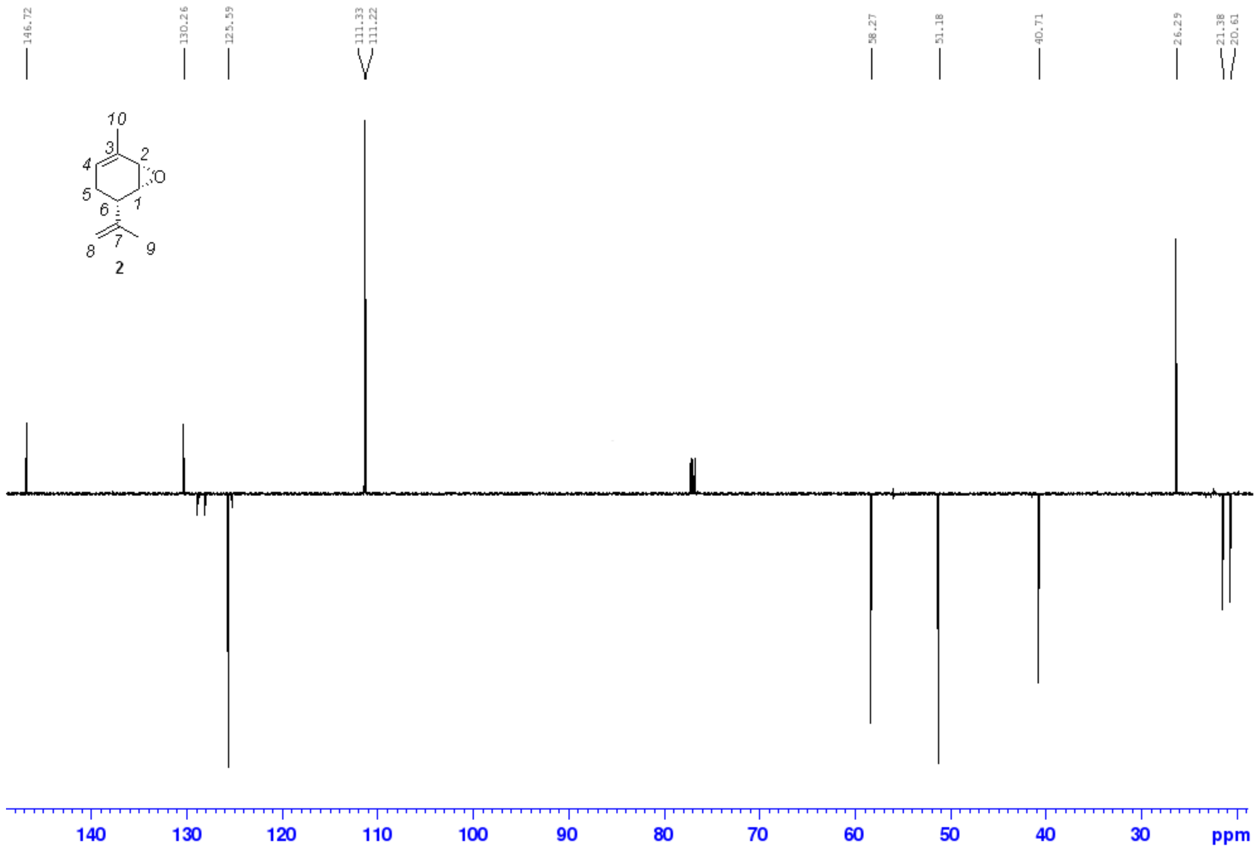

Figure S2: ^{13}C (150 MHz, $CDCl_3$) spectrum of **2**

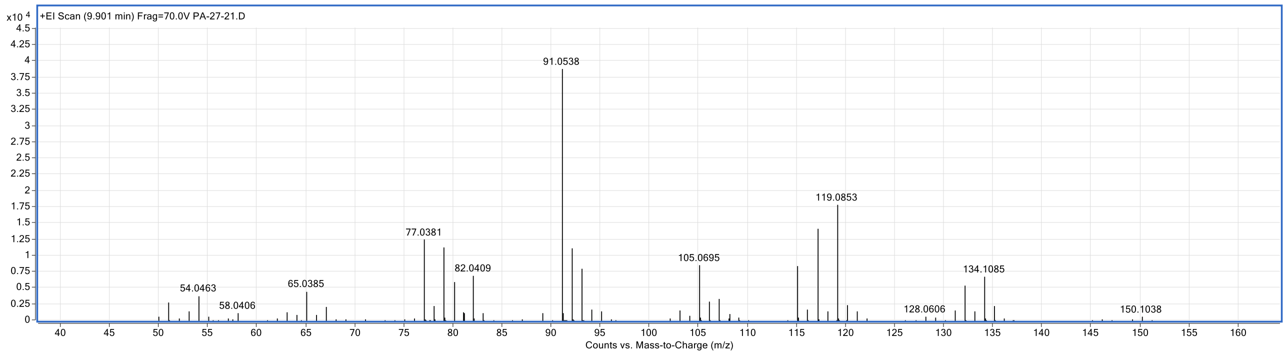

Figure S3: HR-MS spectrum of **2**

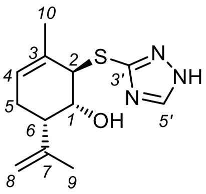

PA96

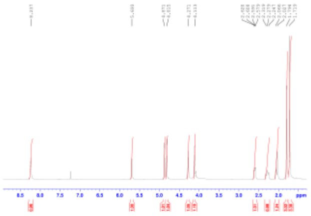

Figure S4: ^{1}H (400 MHz, $CDCl_3$) spectrum of PA96

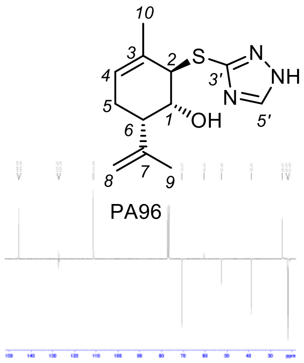

Figure S5: ^{13}C (100 MHz, $CDCl_3$) spectrum of PA96

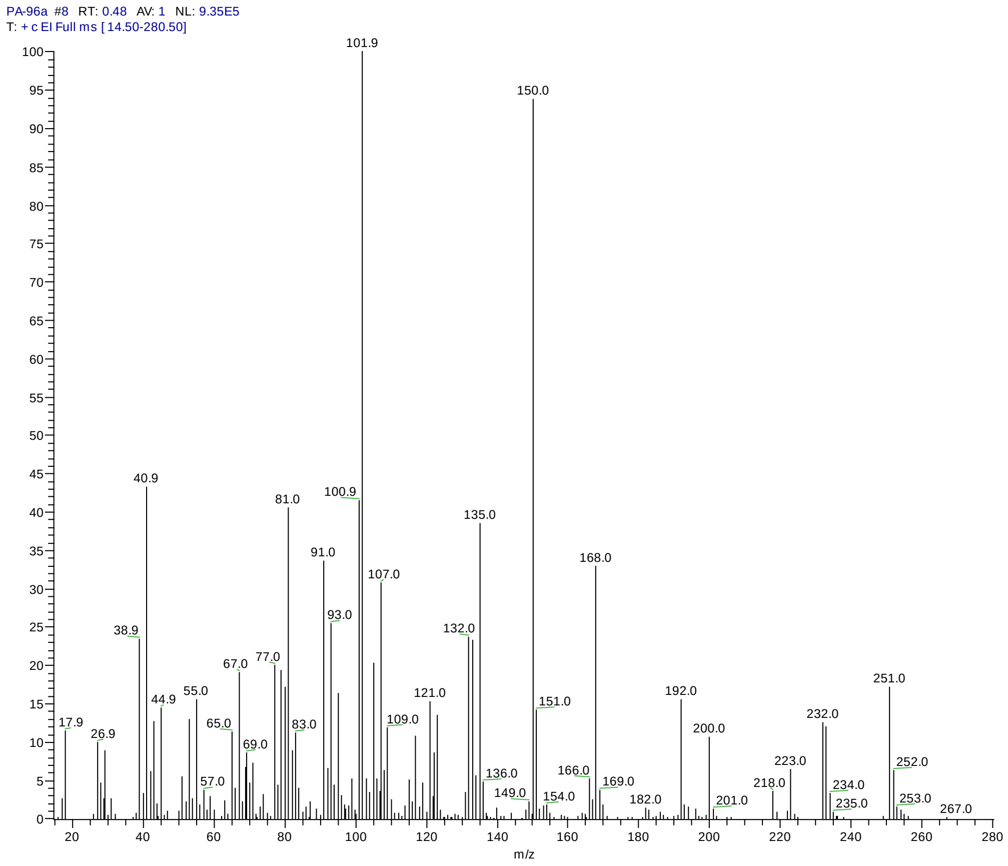

Figure S6: HR-MS spectrum of PA96

Table S1. Results of screening the effect of diol derivatives on naïve and MPP^+-treated dopamine neurons
Support effect >100% – ++++; 100-70% – +++; 69-30% – ++; 29-1% – +; 0% – 0
Inhibition 69-30% – - -; 29-1% – -
Compared to VEH-treated wells

Substance	Structure	Naïve		MPP^+		
		0,1 uM	1 uM	1 nM	10 nM	100 nM
1	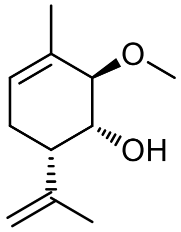	+	-			
2	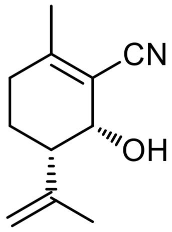	+	+			
3	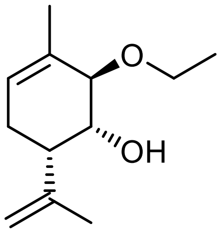	+	++	0	0	+
4	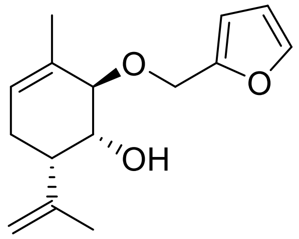	-	-			
5	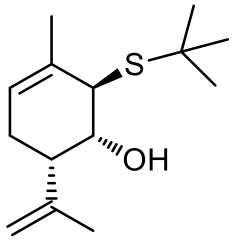	-	-			

PA-96	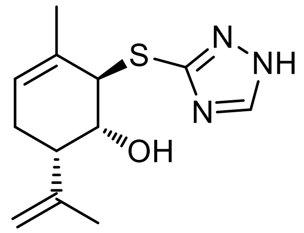	++	+	+++	+	0
7	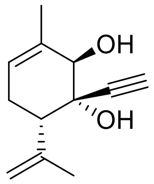	-	-			
8	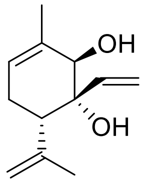	++	+	0	0	0
9	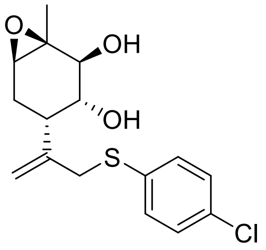	-	-			
10	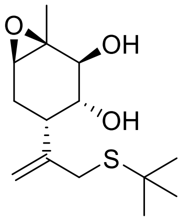	-	-			
11	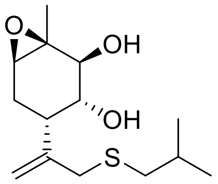	0	- -			
12	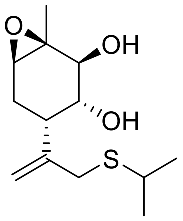	0	+			
13	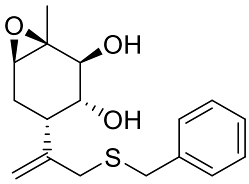	0	-			

14	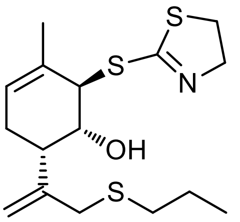	0	+			
15	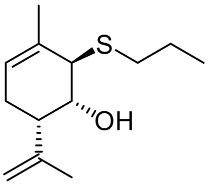	0	-			
16	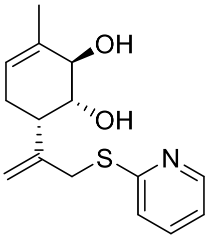	0	+			
17	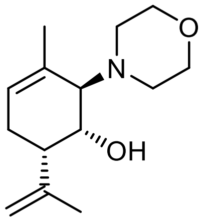	0	-			
18	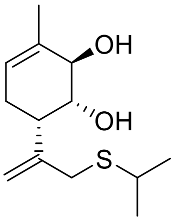	-	+			
19	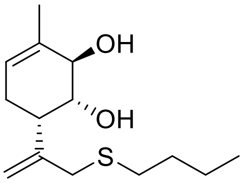	+	+			
20	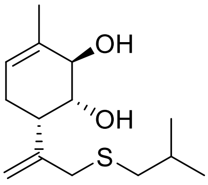	+	+			
